# Editorial for the SEB 2020 special issue ‘dynamic organisation of the nucleus across kingdoms’

**DOI:** 10.1080/19491034.2021.1883294

**Published:** 2021-02-04

**Authors:** David E. Evans

**Affiliations:** Department of Biological and Medical Sciences, Oxford Brookes University, Oxford, UK

This special issue is a collection of papers submitted by authors invited to participate in the 2020 Society for Experimental Biology meeting on the theme of 'Dynamic Organisation of the Nucleus Across Kingdoms', co-organized by Roland Foisner, Philippe Colas, David Evans and Katja Graumann. The conference presentations were postponed to 2021 (https://www.sebiology.org/events/event/seb-antwerp-2021) due to the impact of Covid-19, but these collected papers written in the summer and autumn of 2020 present the cross-kingdom insights and novel findings that were central to the aim of the meeting. The meeting is the 3rd in a series [1, 2] intended to highlight the immense value of sharing knowledge of the nucleus across kingdoms. Here we present a combination of review and original results and methods providing new insights into the field in a landmark year.

Understanding the origins of the structural components of the nucleus underpins many of our efforts to advance understanding of mechanisms and function. This collection of papers provides significant insights – both across kingdoms [3] and in detailed reviews of the current state of knowledge in higher plants [4, 5]. One of the fascinations of studying the dynamic structure of the nucleus is the way in which a range of conserved functions are carried out by such a diversity of lineage-specific components. While a small number of highly conserved proteins point back to their presence in the Last Eukaryotic Common Ancestor, many show a surprising diversification and even functionally conserved proteins show a wide range of structural characteristics. Indeed, from this collection of papers, the reader can only wonder whether the statement of Padilla-Meija et al. [3] that ‘findings suggest a rather surprising level of divergence associated with a structure that, in a very real sense, defines the eukaryotic cell’ is, in fact, an understatement.

While recognizing the limitations imposed by the challenges of defining the nuclear proteome, Padilla-Meija and coworkers [3] provide detailed comparative insights into its evolution using carefully selected data from protozoans to mammals. Through a comparative analysis of previously described datasets from model systems and by expansion of this data, for instance, by searching using queries from *Trypanosoma brucei*, they provide a valuable coverage of nuclear constituents, structure and function, providing insights and a data set of great value for further exploration. Nuclear Envelope Associated (NEA) proteins provide particular challenges. Some are also found in other cellular locations, others are synthesized at the NE; others are multifunctional, with only a small part of their activity at the NE and many have only been characterized in one model organism while their functions in others are uncertain. There is much to be done!

Two other papers in the collection expand the overview of Padilla-Meija to consider advances in knowledge of the plant nuclear proteome and its function. Groves et al. [4] provide a comprehensive survey of the proteins described to date associated with the plant NE, their interactors, locations and functions. Most are lineage-specific, though some proteins (particularly the SUN domain proteins) and many functions of the NE are conserved. Here also, significant divergence is observed in key proteins, even at the species level. While much foundational information has been gained from the model plant *Arabidopsis thaliana*, full understanding requires data from a wide range of species; this is provided in this review and in the review by Evans et al. [5].

In the second plant review, Evans, Mermet and Tatout [5] seek to make the rapidly expanding knowledge of the plant nucleus available for application in crop science. Just as work on the mammalian nucleus informs our understanding of a number of diseases, so also work on plants provides opportunity for meeting global challenges in food production. Knowledge of chromatin structure and positioning and its effect on gene expression, of signaling at the NE, of the role of plant NE proteins in responses to biotic and abiotic stress all provide opportunities for the selection of enhanced traits in plants.

Two additional papers complete the Special Issue. In an example of the highly specialized functions undertaken by members of nuclear protein families, Moser et al. [6] build on previous work on the role of plant KASH-domain proteins in the tip-growing pollen tube. They show for the first time that the active positioning of the vegetative nucleus in proximity to the pollen tube tip is required for response to reactive oxygen species (ROS) and for Ca^2+^ signaling; this is interestingly directly associated with the role of KASH proteins of the WIP family in nuclear positioning. In the second paper, Dubos et al. [7] build on work to develop automated systems to analyze the shape, structure and function of the nucleus through the publicly available ImageJ plug-in NucleusJ 2.0. Developed initially for application in plants, this plug-in is appropriate for the analysis of images of the nucleus across kingdoms.

Taken together, these papers illustrate the great value, encompassed in the principles of these special issues and the meetings that underpin them, of sharing knowledge and methods across kingdoms. From highly conserved proteins, structures and functions, to the highly divergent and sometimes highly specialized systems under study, these papers provide a valuable resource for anyone studying nuclear dynamics and function.

## References

[1] Evans D, Meier I, Graumann K. Editorial for the SEB Brighton special issue: dynamic organization of the nucleus. Nucleus. 2017;8(1):1. 28125395 10.1080/19491034.2016.1264553 PMC5287207

[2] Evans DE, Graumann K, Foisner R. Editorial for the SEB florence special issue: functional organisation of the nuclear periphery. Nucleus. 2019;10(1):167–168. 31345104 10.1080/19491034.2019.1643140 PMC8871612

[3] Padilla-Mejia NE, Makarov AA, Barlow LD, Butterfield ER, Field MC. Evolution and diversification of the nuclear envelope. Nucleus. 2021. doi:10.1080/19491034.2021.1874135 PMC7889174 33435791

[4] Groves NR, Biel A, Moser M, et al. Recent advances in understanding the biological roles of the plant nuclear envelope. Nucleus. 2020;11(1):330–346. 33161800 10.1080/19491034.2020.1846836 PMC7746247

[5] Evans DE, Mermet S, Tatout C. Advancing knowledge of the plant nuclear periphery and its application for crop science. Nucleus. 2020;11(1):347–363. 33295233 10.1080/19491034.2020.1838697 PMC7746251

[6] Moser M, Kirkpatrick A, Groves NR, et al. LINC-complex mediated positioning of the vegetative nucleus is involved in calcium and ROS signaling in Arabidopsis pollen tubes. Nucleus. 2020;11(1):149–163. 32631106 10.1080/19491034.2020.1783783 PMC7529407

[7] Dubos T, Poulet A, Gonthier-Gueret C, et al. Automated 3D bio-imaging analysis of nuclear organization by NucleusJ 2.0. Nucleus. 2020;11(1):315–329. 33153359 10.1080/19491034.2020.1845012 PMC7714466

